# Trends in asymptomatic STI among HIV-positive MSM and lessons for systematic screening

**DOI:** 10.1371/journal.pone.0250557

**Published:** 2021-06-24

**Authors:** Eric Farfour, Svetlane Dimi, Olivier Chassany, Sébastien Fouéré, Nadia Valin, Julie Timsit, Jade Ghosn, Claudine Duvivier, Martin Duracinsky, David Zucman, on behalf of the DRIVER study group

**Affiliations:** 1 Service de Biologie Clinique, Hôpital Foch, Suresnes, France; 2 Centre de Vaccinations Internationales et Médecine de Voyage, Creil, France; 3 Patient-Reported Outcomes Research, Sorbonne Paris Cité, Université Paris-Diderot, Paris, France; 4 Unité de Recherche Clinique en Economie de la Santé, Hôpital Hôtel-Dieu, Assistance Publique Hôpital de Paris, Paris, France; 5 Centre for Genital and Sexually Transmitted Diseases, Dermatology Department, APHP Hospital Saint-Louis, Paris, France; 6 Hôpital Saint Antoine, Service des Maladies Infectieuses, Paris, France; 7 Assistance Publique-Hôpitaux de Paris, Service des Maladies Infectieuses et Tropicales, Groupe Hospitalier Paris Nord Val de Seine, Site Bichat-Claude Bernard, Paris, France; 8 INSERM UMR 1137 IAME, PRES Sorbonne Paris Cité, Université Paris Diderot, Paris, France; 9 APHP-Hôpital Necker-Enfants Malades, Service de Maladies Infectieuses et Tropicales, Centre d’Infectiologie Necker-Pasteur, Paris, France; 10 IHU Imagine, Paris, France; 11 Institut Cochin, CNRS 8104, INSERM U1016, RIL Team: Retrovirus, Infection and Latency, Université de Paris, Paris, France; 12 Institut Pasteur, Centre Médical de l’Institut Pasteur, Paris, France; 13 Médecine Interne et Immunologie Clinique, Hôpital Bicêtre, Assistance Publique Hôpital de Paris, Paris, France; 14 Réseau Ville-Hôpital, Service de Médecine Interne, Hôpital Foch, Suresnes, France; Agencia de Salut Publica de Barcelona, SPAIN

## Abstract

The burden of STIs is particularly high in HIV-infected MSM patients. A recent increase in STIs prevalence has been noticed in the US and western European countries. We aim to assess trends in asymptomatic STIs following the publication of recommendations for STIs screening, i.e. Chlamydia (CT) and gonorrhea (NG). Seventeen centers located in the Paris area participated in the study. All asymptomatic HIV-infected MSM patients attending a follow up consultation were proposed to participated in the study. Asymptomatic patients were included over 2 periods: period 1 from April to December 2015 and period 2 from September to December 2017. Etiologic diagnosis of STIs including hepatitis B, C, syphilis, was performed using a serological test, including a non-treponemal titer with a confirmatory treponemal assay for syphilis. CT and NG were screened using a nucleic acid amplification test (NAATs) on 3 anatomical sites, i.e. urine, rectal and pharyngeal. Overall, 781 patients were included: 490 and 291 in periods 1 and 2 respectively. Asymptomatic CT, NG, and syphilis were diagnosed in 7.5%, 4.8% and, 4.2% respectively. The rate of patients having a multisite asymptomatic infection was 10.2% and 21.1% for CT and NG respectively. The most frequently involved anatomical sites for CT and NG asymptomatic infections were anorectal (66.1% and 55.2% respectively) and pharyngeal (47.4% and 60.5% respectively). CT and NG asymptomatic infection increased by 1.3- and 2-fold respectively between the two periods while syphilis decreased by 3 folds. Our results encourage to reconsider multisite screening for CT and NG in asymptomatic HIV positive MSM as the yield of screening urinary samples only might be low. Despite the more systematic STI screening of asymptomatic HIV positive MSM the prevalence of STI is increasing in MSM in France. Therefore, this strategy has not led to alter CT and NG transmission. The decrease of syphilis might involve self-medication by doxycycline, and the intensification of syphilis screening.

## Introduction

Sexually transmitted diseases (STIs) are a major public healthcare concern in sexually active patients and mostly in men who have sex with men (MSM) and HIV-positive patients. Trends in STIs prevalence are variable worldwide depending on health-care facilities, STIs prevalence, patient’s behavior, STI screening by providers, and STI treatment of infected persons and their partners [1, 2]. Furthermore, STI trends in HIV-positive MSM could have be influenced by the “undetectable = untransmittable” (U = U) campaigns. In high-income countries such as France, the burden of STIs is particularly high in MSM and HIV-infected patients [3, 4]. In France, some STIs such as lymphogranuloma venereum (LGV) are diagnosed almost exclusively in MSM, who represented 95% of all cases reported in 2016. MSM also accounted for 81% of early syphilis cases reported that same year. The number of syphilis cases raised by two-fold between 2010 and 2016, even if stability has been noticed between 2015 and 2016. Likewise, gonorrhea (NG) has increased by 127% between 2014 and 2016. Furthermore, a large outbreak of hepatitis A among MSM occurred in Europe in the middle of the 2010s [5, 6]. Moreover, the increased prevalence of STIs occurs in the context of recurrent shortages of medication such as the hepatitis A vaccine or Benzylpenicillin [5, 7].

Consequently, healthcare authorities have recently recommended screening for asymptomatic STI in all sexually active individuals and especially in MSM. NG and Chlamydia (CT) should be screened at 3 anatomical sites, i.e. urine, pharyngeal and anorectal, every 3 months [8–11].

In the context of the publication of European recommendations for STIs screening, the present study aimed at assessing the trends of asymptomatic STIs prevalence in HIV-positive MSM attending a routine HIV visit [10].

## Materials and methods

This work is a part of the DRIVER project, a prospective multicentric project with two periods of inclusions designed to validate a STIs predictive score. It was approved by the French Ethics Committee (IDRCB 2014-A01358-39) and registered at ClinicalTrials.gov (ID number NCT02413632). Seventeen centers located in the Paris area participated in the study (**Table 1**). All HIV-positive MSM patients, attending their biannual follow-up visits, and able to respond to questionnaires in French were consecutively proposed to participate in the study. Symptomatic patients with STI clinical symptoms or skin rash were excluded. Patients were given a unique identifier and included over one of two periods: i) period 1 from April to December 2015 and; ii) period 2 from September to December 2017. All volunteers provided written informed consent.

**Table 1:** Number of patients included by each center.

Centre	Hospital type	Period 1	Period 2
Hôpital Foch (Suresnes)	PPE	76	17
CHU Bicêtre (Kremlin-Bicètre)	Teaching hospital	46	39
CHU Saint Louis (Paris)	Teaching hospital	24	0
CHU Louis Mourier (Colombes)	Teaching hospital	17	1
Hôpital Européen George Pompidou (Paris)	Teaching hospital	53	24
Institut Mutualiste Montsouris (Paris)	PPE	25	6
CHU Necker (Paris)	Teaching hospital	45	15
Grand Hôpital de l’Est Francilien (Marne-la-Vallée)	General Hospital	20	8
Institut hospitalier Franco-Britannique (Levallois-Perret)	PPE	18	8
CHU Hôtel Dieu (Paris)	Teaching hospital	51	30
CHU Ambroise Paré (Boulogne)	Teaching hospital	26	6
CHU Raymond Poincaré (Garches)	Teaching hospital	50	31
CH Victor Dupouy (Argenteuil)	General hospital	39	10
CHU Saint Antoine (Paris)	Teaching hospital	0	71
CHI Poissy—Saint Germain (Saint-Germain-en-Laye)	General hospital	0	9
CH A. Mignot (Le Chesnay)	General hospital	0	5
CHI de Villeneuve Saint Georges (Villeneuve Saint-Georges)	General hospital	0	11
**Total**		**490**	**291**

STIs screening was performed as recommended by guidelines and manufacturer:

- a Nucleic Acid Amplification Test (NAAT) performed on 3 anatomical sites (first-catch urine sample, rectal swab, and pharyngeal swab) for NG and CT;
- a serological test including a non-treponemal (RPR or VDRL) and a treponemal test (TPHA or chemiluminescence assay detecting total antibody to *T*. *pallidum*) for syphilis. An incident syphilis cases was considered in case of a new positivity or a 4-fold increase in the non-treponemal title.
- serological tests for hepatitis B and C were retrieved from the patients’ records.

The NAAT and the serology were performed by the respective laboratories of each participant center. The laboratory technicians were blinded as to study participation.

CD4+ count and HIV viral load were assessed at the inclusion visit.

Socio-demographic and clinical data including age, sex, date of HIV diagnosis, past history of STI, and sexual behavior were collected using a self-administered questionnaire as previously described [12].

Statistical analyses were performed using SSPS software (IBM, Armonk, United-States). Categorical variables were compared using the Fisher’s exact test.

## Results

### Patients’ characteristics

Seventeen centers participated in the study, of which 13 and 16 included patients during the first and the second period respectively (**Table 1**). Overall, 781 patients were enrolled: 490 during the first period and 291 during the second one.

There were no significant differences in socio-demographic characteristics and sexual behavior between patients included in each period (**Table 2**). Their median age was 47, with a median duration of HIV-infection around 12 years (13.00 vs 11.55; *P = 0*.*66*) and a CD4+ count above 500/mm^3^ in around 75% (78.6% vs 74.1%; *P = 0*.*22*). All patients were treated by antiretrovirals (ARV) in group 1 versus 95.9% in group 2 (*P<0*.*01*). HIV viral load was not detectable in 93.47% and 92.78% of the patients included in period 1 and 2 respectively (*P = 0*.*77*). The non-use of condom over the past 6 months was reported by 42.6% and 40.0% of the patients included in periods 1 and 2 respectively (*P = 0*.*68*).

**Table 2:** Characteristics of patients, and history of reported STI.

	First period (n = 490)	Second period (n = 291)	p
Median age (years)	47 [40–54]	47 [39–55]	0.81
Median duration of HIV infection (years)	13 [5.91–22.30]	11.55 [4.53–23.38]	0.67
Median CD4+ count	679 [526–894.75]	634 [497–820.5]	0.70
Patient with CD4+ > 500/mm^3^	385 (78.57%)	217 (74.11%)	0.22
HIV viral loads not detectable	458 (93.47%)	270(92.78%)	0.77
ARV treatment	490 (100%)	279 (95.9%)	**<0.01**
Median duration of ARV treatment (years)	9.91 [4.26–17.97]	8.36 [3.67–19.38]	**0.03**
History of STI	395 (80.6%)	197 (67.7%)	**<0.01**
• Hepatitis A	31 (6.3%)	7 (2.4%)	**0.02**
• Herpes	62 (12.6%)	15 (5.1%)	**<0.01**
• Syphilis	220 (44.9%)	121 (41.6%)	0.37
• *C*. *trachomatis*	56 (11.4%)	32 (11.0%)	0.85
• *N*. *gonorrheae*	112 (22.9%)	50 (17.2%)	0.06
• Proctitis	56 (11.4%)	28 (9.6%)	0.43
• Condyloma	178 (36.3)	79 (27.1)	**<0.01**
• Anal condyloma	172 (35.1%)	75 (25.8%)	**<0.01**
• Genital condyloma	21 (4.3%)	11 (3.8%)	0.73
• Buccal condyloma	3 (0.6%)	3 (1.0%)	0.68
Sexual Behavior			
• no use of condoms	195 (42.6%)	115 (40.0%)	0.68
• Poppers use	188 (39.6%)	103 (35.9%)	0.51
• Viagra^®^ use	84 (17.7%)	47 (16.3%)	0.51

Overall, 75.8% of all patients reported a previous history of STIs (**Table 2**). Most frequent STIs were syphilis (43.6%), anal condyloma (31.6%) and, NG (20.7%), while hepatitis A was reported by only 4.8%. The rate of patients reporting a previous history of STIs was significantly higher in period 1 than in period 2 (80.6% vs 67.7%; *P< 0*.*01*) (**Table 2**). These trends are significant for 3 STIs: symptomatic hepatitis A (6.3% vs 2.4%; *P = 0*.*02*), herpes (12.6% vs 5.1%; *P<0*.*01*) and anal condyloma (36.3% vs 27.1%; *P<0*.*01*). It almost achieved significance for NG (22.9% vs 17.2%; *P = 0*,*06*). A similar but not significant trend was noticed for all other STI’s.

### Chlamydia and gonorrhea

Overall, at least a bacterial STI (CT, NG or Syphilis) was diagnosed in 11.6% and 15.9% of all patients enrolled in period 1 and 2 respectively (*P = 0*.*11*).

**Fig 1:**
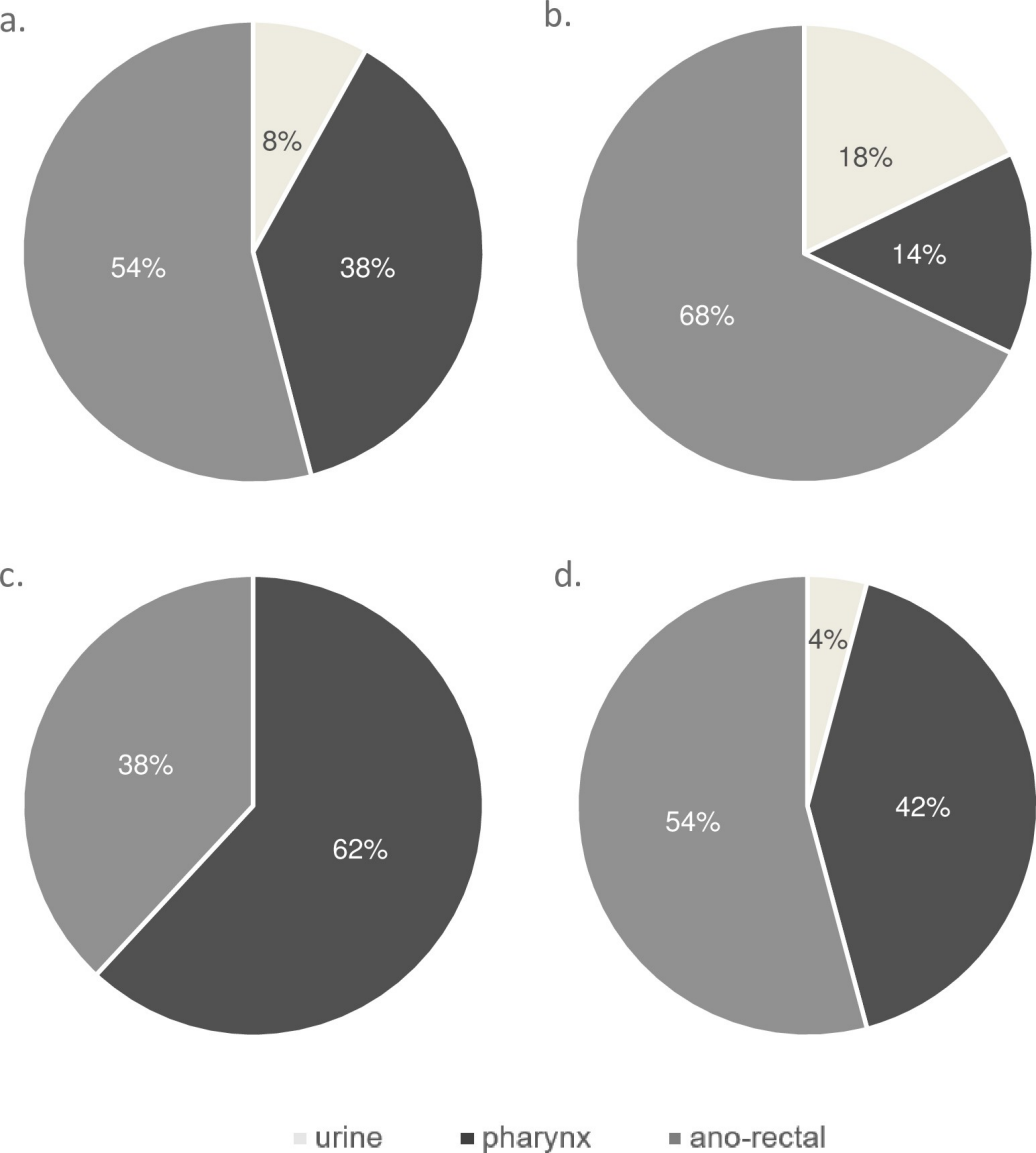
Repartition of anatomical sites positive for *C*. *trachomatis* and *N*. *gonorrheae*. Site of isolation of *C. trachomatis* during period 1 (a.) and 2 (b.) and N. gonorrhea during period 1 (c.) and 2 (d.).

Asymptomatic CT and NG were found in 7.5% and 4.8% of all patients respectively. For both CT and NG most of the patients had a single positive anatomical site (89.8% and 78.9% respectively), but a higher proportion of NG was recovered from 2 anatomical locations (CT: 13.6% vs NG: 21.0%; *P<0*.*01*) (**Table 3**). No pathogens were positive on three anatomical sites. Both CT and NG were mostly recovered from anorectal samples (CT: 66.1% vs NG: 55.3%; *P = 0*.*38*) (**Fig 1**). NG was significantly more often present than CT in pharyngeal samples (CT: 30.5% vs NG: 60.5%; *P<0*.*01*). In contrast, CT was more frequently positive in urine samples (CT: 13.5% vs NG: 2.6%; *P<0*.*01*). Overall, CT was identified 5 and 2 times more often in anorectal and pharyngeal samples respectively than in urinary samples. NG was almost never isolated from urinary samples.

**Table 3:** Asymptomatic co-infection screened at inclusion.

	First period (n = 490)	Second period (n = 291)	P
Hepatitis B	21 (4.30%)	11 (3.78%)	0.85
Hepatitis C	54 (11.06%)	23 (7.90%)	0.17
Active syphilis	28 (5.71%)	5 (1.68%)	**0.01**
*C*. *trachomatis*	33 (6.73%)	26 (8.96%)	0.27
Nb of site of isolation			
• 1 location	29 (87.8%)	24 (92.3%)	0.24
• 2 locations	4 (12.2%)	2 (7.7%)	1
• 3 locations	0	0	
Site of isolation			
• urine	3 (0.61%)	5 (1.72%)	0,16
• pharynx	14 (2.86%)	4 (1.37%)	0,22
• ano-rectal	20 (4.08%)	19 (6.52%)	0,17
*N*. *gonorrhoeae*	18 (3.67%)	20 (6.89%)	0.06
Nb of site of isolation			
• 1 location	15 (83.3%)	15 (75.0%)	0.18
• 2 locations	3 (16.7%)	5 (25.0%)	0.16
• 3 locations	0	0	
Site of isolation			
• urine	0	1 (0.34%)	0,37
• pharynx	13 (2.65%)	10 (5.24%)	0,52
• ano-rectal	8 (1.63%)	13 (4.47%)	0,02
At least a bacterial STI	11.6%	15.9%	0.11

CT prevalence increased by 30% between periods 1 and 2. Anorectal and urinary samples tended to be more frequently positive in period 2 while the rate of positivity of pharyngeal samples tended to decrease. NG prevalence increased two-fold between the 2 periods of study. Anorectal location of NG was significantly more frequent during period 2 (4.47% vs 1.63%; *P = 0*.*02*) and pharyngeal location also trends to increase between the two periods (5.24% vs 2.65%; *P = 0*.*52*).

### Hepatitis B, hepatitis C, and syphilis

Overall, 32 (4.1%) patients had chronic hepatitis B and 77 (9.8%) had a positive serology for hepatitis C (**Table 3**). Chronic hepatitis B prevalence remains stable between the two-period study (P1: 4.30% vs P2: 3.78%; *P = 0*.*85*), while hepatitis C tended to decrease from 11.06% to 7.90% (*P = 0*.*17*).

Overall, early syphilis was detected in 4.2% patients. Its prevalence fell from 5.71% to 1.68% between periods (*P = 0*.*012*).

## Discussion

The main finding of this work is a recent and rapid change in asymptomatic STIs prevalence among HIV-positive MSM living in the Paris area. Indeed, we report a significant decrease of syphilis prevalence and an increase in asymptomatic CT and of NG infections (which almost reach significance for the latter).

CT is the main STI in the general population in France. Its prevalence is increasing in all categories of the population [3]. However, 2 findings characterized MSM: i) serovar L causing *Lymphogranuloma venerum* (LGV), an emerging infectious disease in Western Europe and North America, is almost exclusively found in this population [3, 13], nevertheless, CT serovar were not identified in the present study. And; ii) pharyngeal and anorectal asymptomatic infection are more frequent than asymptomatic urinary infections [14–17].

Multisite screening might enhance the identification of asymptomatic CT infected patients. However, in the present study, the 3 anatomical sites display a variable rate of positivity, urinary samples being the least frequently positive sample. In a monocentric study in Germany involving 296 asymptomatic HIV-positive MSM, CT was detected in 7.3%, 1.7% and 1.0% of anorectal, pharyngeal and, urethral samples respectively [14]. NG was detected in 4.5%, 2.0% and, 1.4% respectively. de Vrieze *et al*. reported a CT prevalence of 3.32% and 8.30% in urethral and anorectal specimens of MSM attending a STI clinic in Amsterdam [15]. However, some of these patients were symptomatic. In France, CT prevalence in pharyngeal, anorectal, and urine samples was assessed to 1%, 8% and, 3% in a cohort of 116 MSM (of which 99 HIV-positive) living in Paris area [16]. In contrast, the rate of positivity of urethral samples reaches 29.1% in a cohort of 346 asymptomatic MSM living in Thailand (versus 17.6% and 17.0% for pharyngeal and anorectal samples respectively) [18]. This suggests that, screening strategies should perhaps be adapted regarding local epidemiology. Considering that asymptomatic patients have a low rate of positive urinary samples in comparison to anorectal and pharyngeal samples, our results suggest stopping the screening of CT and NG in urinary samples in France and other western European countries with similar prevalence of CT and NG [17].

Furthermore, three anatomical site screening of CT and NG for asymptomatic STI was implemented in all centers at the beginning of the study. The relatedness between the increased prevalence of both CT and NG in period 2 and the implementation of multisite screening remains to be determined. Despite the patients enrolled in periods 1 and 2 were different, a change in patients’ risk behavior, which could explain a rise in STIs prevalence, was not found, e.g. the rate of condom use was not different, 43% in the period 1 and 40% period 2 (*p = 0*,*68*). One objective of the strategy of systematic screening of asymptomatic STI recommended by WHO and local Health-care authority was to reduce STIs transmission [8, 10, 19]. However, there is no evidence that this strategy reduces the transmission of these pathogens [20]. And, as for some pathogens such as *N*. *meningitidis*, we could hypothesis that carriage strains are genetically different from infectious strains [21]. Further studies are therefore needed in order to understand the potential pathogenic role of asymptomatic strains and also the possible quantitative role of infectious pathogen load in the occurrence of symptoms. As a consequence of systematic screening, antibiotics use is increasing in the sexually active population, leading to a potential impact on micro-organisms resistance [22]. Consequently, the risks and benefits of CT and NG screening should be assessed having in mind the large use of antibiotics in the sexually active population.

Surprisingly, syphilis prevalence fell 3-fold between the 2 periods of study. This trend is opposite to most reports. In France, syphilis reported cases have consistently increased since the early 2000s [1, 3, 23] though the prevalence of early infection remained stable between 2015 and 2016 [3]. The significant opposite trend reported in the present cohort of HIV-positive MSM patients could be explained by enrollment bias. First, one third of patients were included in period 2 by 4 centers that had not participated in period 1. Then, a previous history of STI was more frequently reported by patients enrolled in period 1. However, CT and NG prevalence increased as expected. Therefore, both biases would probably have no impact on syphilis prevalence. As a similar rate of patients included in periods 1 and 2 reported a previous history of syphilis, it is unlikely that immunity factors could explain such a difference in active syphilis rate–besides, it is common knowledge that previous syphilis does not induce protective immunity against re-infection. Over the past decade, the salient features of the evolution in syphilis prevalence were related to change in: i) the perception of sexual risk, ii) the diagnosis and immediate treatment of HIV (Treatment as Prevention or TasP) and other STIs and, iii) perception of sexual behavior risk [24–27]. PrEP for HIV prevention, has been in use for several years, and, recently a similar strategy using doxycycline has been assessed to prevent bacterial [28, 29]. Doxycycline PrEP was associated with a significant reduction in syphilis and CT but not NG in HIV- MSM on PrEP, and studies are currently being conducted to evaluate the impact on STIs in HIV+ MSM and to confirm the results in HIV- MSM on PrEP. It may be hypothesized therefore that some patients could have taken antibiotics to prevent STIs even in the absence of recommendations. Indeed, in the United-Kingdom, in 106 patients followed at an HIV Prep consultation, 8% self-medicated with antibiotics in order to prevent bacterial STIs [30]. Such could have been the case for patients enrolled in period 2 (though our study’s design did not allow us to investigate this issue).

STIs are still of major health concern in HIV-positive MSM, but several recent changes in their prevalence are occurring. Today, it is demonstrated that there is no risk of transmission of HIV from an HIV-positive person with consistent undetectable viral load to an HIV negative person during sexual intercourse, regardless of gender or sex. This treatment as prevention (TasP) message has been a part of a public campaign as “undetectable = untransmittable” (U = U) [31, 32]. For the French HIV positive MSM, ‘having a sexual partner’ and condomless sex, both increased in frequency between 2000 and 2017 [33]. The NG prevalence are increasing in this population and anorectal and pharyngeal sites are probably a major reservoir of these pathogens. These results encourage to review the current strategy of multi-site screening by stopping considering urine samples or even to stop performing CT and NG screening. Molecular and serological characterization will perhaps provide more insight into CT and NG natural history, pathogenesis and transmission.

Our study might not be representative of HIV-positive MSM as all patients enrolled lived in the Paris area. We could not exclude the screening recommendations are followed by all centers in France. Furthermore, some clinics only participated in one of the 2 time periods, and a small number of patients were enrolled from some others. Sexual behavior and antibiotic consumption in the past previous year could probably reveal another insight into the trend of STI prevalence in HIV-positive MSM.

## Conclusion

In conclusion, in asymptomatic HIV positive MSM attending their routine consultation in Paris area, a systematic screening reveals a bacterial asymptomatic STI in at least 11% of the patients. Our results encourage to reconsider the interest of systematic multisite screening for CT and NG in asymptomatic patients as the positive screening in urinary samples are low and this systematic screening has not led to disrupt CT and NG transmission. Assessing the impact of the current strategy of STIs screening is therefore needed. On the contrary, the prevalence of asymptomatic syphilis has decreased between 2015 and 2017. The reasons for this change are probably multifactorial involving self-medication by doxycycline, implementation, and generalization of active screening in the middle 2010s.
